# Midterm survival and risk factor analysis in patients with pyogenic vertebral osteomyelitis: a retrospective study of 155 cases

**DOI:** 10.3389/fsurg.2024.1357318

**Published:** 2024-05-21

**Authors:** Melanie Schindler, Nike Walter, Jan Reinhard, Stefano Pagano, Dominik Szymski, Volker Alt, Markus Rupp, Siegmund Lang

**Affiliations:** ^1^ Department of Trauma Surgery, University Hospital Regensburg, Regensburg, Germany; ^2^ Department of Orthopaedic Surgery, University Hospital of Regensburg, Asklepios Klinikum Bad Abbach, Bad Abbach, Germany

**Keywords:** vertebral osteomyelitis (VO), mortality, pathogens, spine infection, comorbidities

## Abstract

**Background:**

Pyogenic vertebral osteomyelitis (VO) represents a clinical challenge and is linked to substantial morbidity and mortality. This study aimed to examine mortality as well as potential risk factors contributing to in-hospital mortality among patients with VO.

**Methods:**

This retrospective analysis involved patients receiving treatment for VO at University Regensburg in Germany from January 1, 2000, to December 3, 2020. It included in-hospital mortality rate, comorbidities and pathogens. Patients were identified using ICD-10 diagnosis codes: M46.2, M46.3, M46.4, and M46.5. Kaplan–Meier probability plots and odds ratios (OR) for mortality were calculated.

**Results:**

Out of the total cohort of 155 patients with VO, 53 patients (34.1%) died during a mean follow-up time of 87.8 ± 70.8 months. The overall mortality was 17.2% at one year, 19.9% at two years and 28.3% at five years. Patients with congestive heart failure (*p* = 0.005), renal disease (*p* < 0.001), symptoms of paraplegia (*p* = 0.029), and sepsis (*p* = 0.006) demonstrated significantly higher overall mortality rates. In 56.1% of cases, pathogens were identified, with *Staphylococcus aureus (S. aureus)* and other unidentified pathogens being the most common. Renal disease (OR 1.85) and congestive heart failure (OR 1.52) were identified as significant risk factors.

**Conclusion:**

Early assessment of the specific risk factors for each patient may prove beneficial in the management and treatment of VO to reduce the risk of mortality. These findings demonstrate the importance of close monitoring of VO patients with underlying chronic organ disease and early identification and treatment of sepsis. Prioritizing identification of the exact pathogens and antibiotic sensitivity testing can improve outcomes for patients in this high-risk group.

## 1 Introduction

Musculoskeletal infections present a significant challenge in orthopedic trauma surgery, particularly when involving the spine (1). These infections can occur as pyogenic vertebral osteomyelitis (VO), encompassing spondylitis, discitis and vertebral osteomyelitis (2, 3), requiring hospitalization for the affected patients (4, 5). The predominant cause are infectious organisms, with hemolytic *Staphylococcus aureus (S. aureus)* being the most frequent (6). VO is a relatively rare form of osteomyelitis, making up only about 3% to 5% of all infectious bone diseases (4).

Epidemiological studies have consistently indicated a rising incidence of VO and spondylodiscitis in Europe, underscoring the ongoing challenge for healthcare systems (7–9). In Germany the highest in-hospital mortality rate was recorded at 64.7 per 1,000 patients and intensive care unit (ICU) treatment was documented in 27.7% of cases (8). Intriguingly, in contrast to diagnoses such as major amputations, VO demonstrates comparatively lower mortality rates (10, 11). The treatment of elderly patients, who are particularly vulnerable to these infections, is becoming increasingly important due to epidemiological development and the growing prevalence of comorbidities in an aging population (12). Furthermore, secondary diagnoses contributing to frailty, such as liver cirrhosis, congestive heart failure, and kidney insufficiency requiring dialysis, have been associated with a higher risk (13). VO treatment also affects patients’ quality of life. Remarkably, even seven years on average following the completion of VO treatment, patients continue to experience diminished quality of life compared to the reverence population (14).

The hidden characteristics of these infections, especially when triggered by low-virulence pathogens, often result in delayed diagnoses, resulting in significant morbidity and mortality (15, 16). Notably, infections associated with *coagulase-negative staphylococci (CoNS)* are gaining importance in this context (17). Recent literature has summarized updated diagnostic and therapeutic approaches for treatment (18, 19). However, significant heterogeneity persists in reports on the epidemiology of these conditions, with limited analyses of nationwide databases and an incomplete understanding of in-hospital mortality rates (4, 7, 20). The mortality rate ranges between 2% and 12.4% (21–23).

In response to these challenges, this study aims to (1) investigate the mortality rates of patients with VO stratified for comorbidities. Second (2) to identify potential risk factors for in-hospital mortality among VO patients.

## 2 Material and methods

We conducted a retrospective study involving patients treated for VO at a university trauma center in Germany. The study period extended from January 1, 2000, to December 3, 2020. Patients were identified based on specific international disease classification (ICD-10) diagnosis codes, which included M46.2, M46.3, M46.4, and M46.5. We screened patients’ medical records, surgical records, radiological findings, laboratory results, microbiological reports, and histopathological reports to determine eligibility for inclusion in the study. We specifically included patients who had completed their treatment for VO, and this completion was well-documented in their medical charts. We collected patient characteristics, including sex, age, body mass index (BMI) at the time of admission, mortality and details of the treatments received (surgery, revision surgery, and changes in antibiotic regimens) by reviewing electronic medical records. The surgical indications for VO in this retrospective study were based on hospital-internal standard operating procedures (SOPs) and tailored individually for each case. Given the study's retrospective nature, some heterogeneity in surgical indications across cases cannot be excluded. In general indications for surgical treatment of pyogenic spondylodiscitis are sepsis, an epidural abscess, neurological deficits/complications, and instabilities/deformities in the affected motion segment (24). Preservation of vertebral body integrity, development of spinal deformities, and refractory back pain, inadequate patient compliance, and failure of conservative therapy are considered relative surgical indications (25). Segmental kyphosis >15°, vertebral body loss >50%, and/or translation >5 mm are considered instability criteria (26). Obesity is defined as a BMI ≥25. Comorbid conditions were evaluated by acquiring the Charlson Comorbidity Index (CCI) and the age-specific Charlson Comorbidity Index (ACCI) (27). To conduct subgroup analyses, we categorized the causes of VO into two groups: community-acquired vertebral osteomyelitis (CAVO) and healthcare-associated vertebral osteomyelitis (HAVO), as previously defined (16). The criteria were:

I. Onset of symptoms occurring after one month of hospitalization without signs of vertebral osteomyelitis upon admission.
II. Hospital admission within six months prior to symptom onset.
III. Outpatient diagnostic or therapeutic interventions within six months prior to symptom onset (long-term use of a central venous catheter, arteriovenous fistula for hemodialysis, invasive intravascular techniques, urological, gynecological, or gastrointestinal procedures, and skin procedures).
IV. Spondylodiscitis cases not meeting any of the above criteria were classified as CAVO

Exclusion criteria were defined as: tuberculous, fungal, or viral spondylodiscitis, brucella VO, implant-associated VO (IAVO), incomplete dataset, malignant or metastatic disease of the spine.

Further we contacted the patients by phone.

### 2.1 Statistical analysis

Data analysis was performed using SPSS Statistics version 28.0 (SPSS Inc, Chicago, IL, USA). We calculated descriptive statistics for all variables. Continuous variables were presented as mean values and standard deviations. The study presents the frequencies of secondary diagnoses, which were identified through coded data. These frequencies are presented as both the actual numbers and as proportions relative to the total number of cases. To make the data more understandable, these secondary diagnoses were categorized into two groups: comorbidities and complications. Additionally, the coded data related to pathogens was carefully analyzed.

Also, a univariate analysis approach was employed to individually assess each variable for its potential role as a risk factor linked to in-hospital mortality among patients diagnosed with VO. The dataset used for this analysis covered a wide range of patient outcomes, including cases with and without documented in-hospital deaths.

We evaluated a Kaplan-Meier analysis with the follow-ups as endpoints. The statistical significance of the association between each parameter and the recurrence-free survival was performed with the ANOVA test.

For various comorbidities and complications of interest, odds ratios (OR) were calculated. In addition to ORs, lower and upper 95% Confidence Intervals (CI) were determined to provide an estimate of the range within which the true OR is likely to fall with a 95% probability.

A multifactorial analysis was conducted between the laboratory values and factors like blood cultures and clinical sepsis syndrome.

The strength of the statistical relationship between exposure to specific factors and the occurrence of a particular outcome, which in this case is in-hospital mortality, was measured using OR.

To investigate the relationship between in-hospital mortality and each variable, a Chi-square test of independence was performed. A significance level (alpha) of 0.05 was chosen, meaning that a *p*-value less than this threshold would indicate a statistically significant association between the variable and in-hospital mortality. The level of statistical significance was set at *p* < 0.05.

### 2.2 Ethical considerations

This study adhered to the principles outlined in the Declaration of Helsinki and received approval from the local university ethics committee (Institutional Review Board Number 12-218_2-101; Amendment 08/2021). Written informed consent was obtained from all individual participants who were included in the study.

## 3 Results

**Figure 1:**
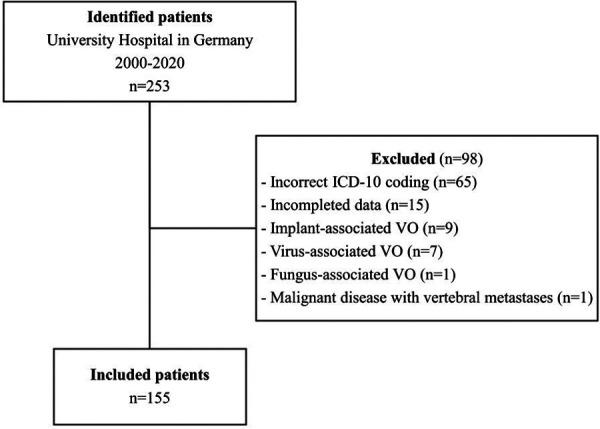
Flow diagram.

Out of the 253 patients with relevant ICD-10 codes, 155 were found to have VO and met the inclusion criteria. Ninety-eight patients were excluded from the study due to the following exclusion criteria (Figure 1): Brucellar or mycobacterial spondylodiscitis, virus- or fungus-associated spondylodiscitis, IAVO, malignant disease with vertebral metastases, incomplete data documentation. In 65 cases, the criteria for diagnosing were not met according to the medical records, so they were excluded from the study due to incorrect ICD-10 coding. Seven patients had tuberculous spondylodiscitis, and nine had IAVO. Fifteen were excluded due to incomplete data, and two were excluded for other reasons (fungal-associated spondylodiscitis and malignant disease of the spine).

So, a total of 155 patients, included 88 men and 67 women, who were treated for VO, were collected in the study between 2010 and 2020 at a University Hospital in Germany. The average age of these patients was 66.7 ± 12.4 (26–93) years.

The mean BMI of these patients was 28.7 ± 7. The mean CCI stood at 4.1 ± 3, while the mean ACCI was recorded at 2.1 ± 1. The mean time from the onset of symptoms to the day of hospitalization was 93.7 ± 290.9 days. The average length of hospital stay for these patients was 36.5 ± 36.3 (1–343) days (Table 1).

**Table 1:** Demografic data.

	All *n* = 155	Mortalities *n* = 53
Mean age (a)	66.7 ± 12	67.7 ± 12
Age >65a	93 (60.0%)	36 (67.9%)
Sex		
Men	88 (56.8%)	35 (66.0%)
Women	67 (43.2%)	18 (34.0%)
Mean BMI (kg/m^2^)	28.7 ± 7	28.6 ± 7
Mean CCI	4.1 ± 3	4.3 ± 2
Clinical symptoms		
Backpain	117 (75,5%)	39 (73.6%)
Fever	25 (16,1%)	8 (15.1%)
Sepsis	34 (21.9%)	19 (35.8%)
Parese	19 (12.3%)	10 (18.9%)
Treatment		
One-stage procedere	69 (44.5%)	26 (49.1%)
Conservative	58 (37.4%)	16 (30.2%)
Mean lenght of hospital stay (days)	36.5 ± 36	64.2 ± 102
Mean follow-up (months)	87.8 ± 71	21.4 ± 26
Location of VO		
Cervical	10 (6.5%)	4 (7.5%)
Thoracic	49 (31.6%)	16 (30.2%)
Lumbal	66 (42.6%)	21 (39.6%)

The majority of patients were treated surgically (*n* = 96, 61.9%). All cervical spondylodiscitis cases (*n* = 10) were managed surgically, while 36 thoracic cases (75%) and 34 lumbar cases (52%) received surgical treatment. Thereof 69 patients (71.9%) got a one-stage procedure. A one-stage operative approach was predominantly undertaken in thoracic spondylodiscitis cases (*n* = 32, 89%; *p* = 0.007). Surgically treated thoracic spondylodiscitis cases frequently received (partial) corpectomy and a vertebral body replacement (VBR; *n* = 28, 57.1%; *p* < 0.001). Patients exhibiting symptoms of paraplegia were significantly more likely to undergo surgical treatment (*n* = 15, 93.8%; *p* = 0.007) and received a VBR (*n* = 11, 68.8%; *p* = 0.002). Surgical intervention was notably more common among patients experiencing paralysis (*n* = 19, 98.5%; *p* = 0.010) and back pain (*n* = 67, 57.8%; *p* = 0.024). Septic patients showed a significantly higher tendency to undergo surgery (*n* = 27, 79.4%; *p* = 0.020). All patients diagnosed with peptic ulcer disease received surgical intervention (*n* = 8; *p* = 0.024). Patients diagnosed with periprosthetic joint infection (PJI) were significantly more likely to received dorsal instrumentation (*n* = 4, 80%; *p* = 0.031). Spondylodiscitis cases associated with *S. aureus* were significantly more likely to undergo surgery (*n* = 34, 75.6%; *p* = 0.030). Skin and subcutaneous tissue infections were the predominant focus in 25.1% of cases, while no identifiable focus was observed in 43.2% of cases (Table 2).

**Table 2:** Primary infection foci.

Infection type	*n* (%)
Skin and subcutaneous tissue	39 (25.1%)
Intraabdominal	22 (14.2%)
Urinary tract	11 (7.1%)
Oral/dental	11 (7.1%)
Prosthetic joints	5 (3.2%)
No identified primary infection foci	67 (43.2%)

### 3.1 Midterm survival of patients with VO

In total, 53 patients, constituting 34.1% of the cohort, did not survive. In the case of 41 patients (26.5%), the age at death was determined through patient records and discussions with family members, averaging 69.21 ± 11.7 years. Additionally, 22 patients (14.2%) passed away in the hospital. The cause of death was identified for 18 patients. Among them, four patients expired due to cardiac arrest, three succumbed to multiorgan failure, seven were attributed to sepsis (accompanied by multiorgan failure), one to drug intoxication, one to respiratory failure, one to graft-vs.-host reaction, and one to fungal pneumonia. Ten patients (6.5%) were lost to follow-up. The mean duration of follow-up was 87.8 ± 70.8 months, ranging from 0 to 214 months.

The one-year survival rate was 82.8%, the two-year survival rate was 80.9%, and the five-year survival rate was 71.7%. The overall mortality was 17.2% at one year, 19.9% at two years and 28.3% at five years.

**Figure 2:**
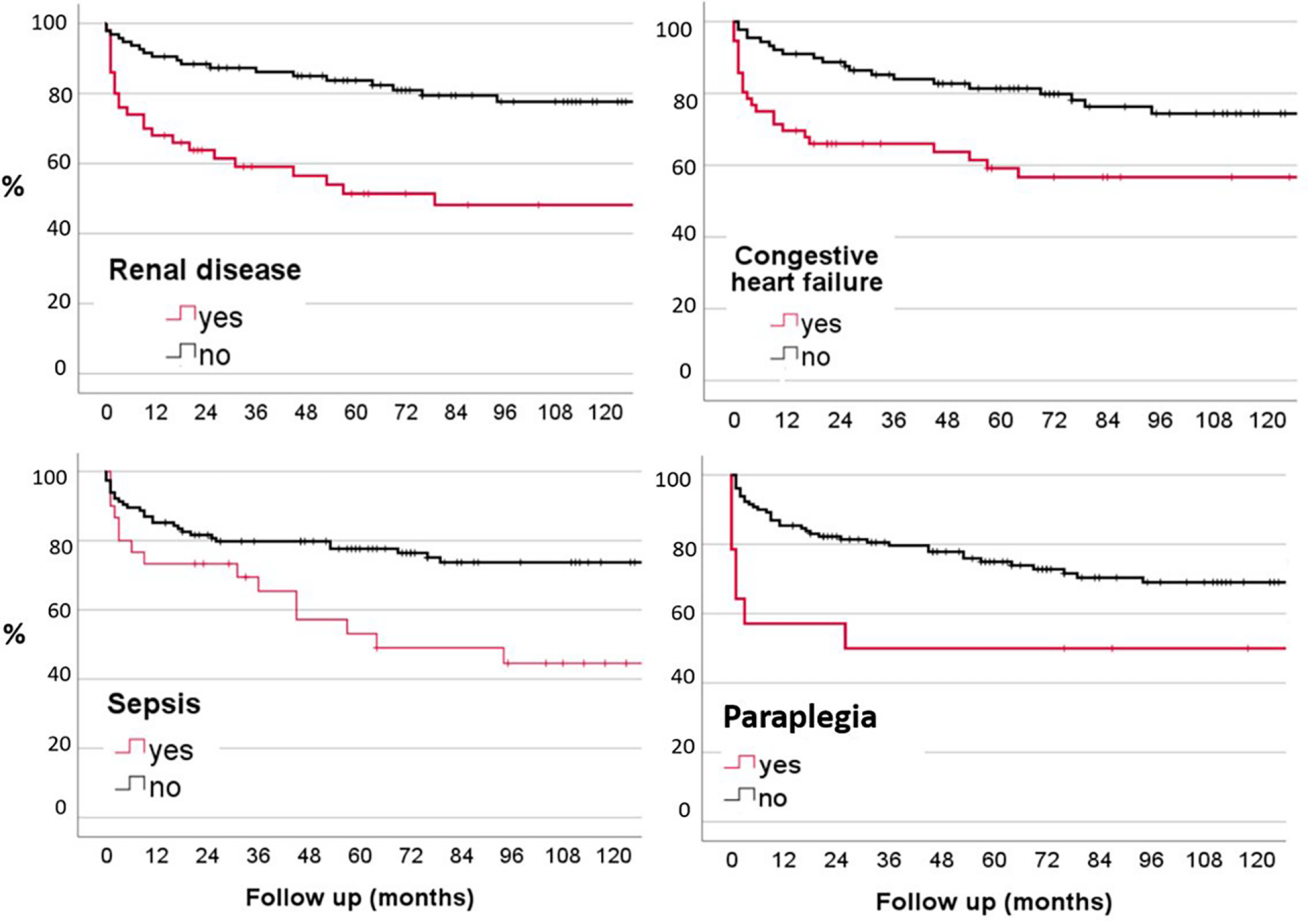
Kaplan-Meier probability plots of mortality related to renal disease, congestive heart failure, paraplegia and sepsis.

Patients who presented with congestive heart failure (38.1%; *p* = 0.005), renal disease (32.9%; *p* < 0.001), symptoms of paraplegia (10.3%; *p* = 0.029), and clinical sepsis syndrome (22%; *p* = 0.006) demonstrated a significant higher rate of mortality than patients without these conditions (Figure 2). Patients with renal disease had a one-year mortality rate of 24%. Otherwise, previous hospitalization, age over 65 years, surgery, abscesses, sex, other comorbidities such as diabetes mellitus, liver disease, were not associated with an elevated mortality rate during the follow-up (Table 3). Half of the patient cohort (*n* = 69) underwent blood culture analysis, and these individuals showed a significantly higher mortality rate (*p* = 0.002) compared to those who did not have blood cultures taken. There is a statistically significant difference between leukocyte values and clinical sepsis (*p* = 0.032). However, there is no other statistically significant difference between CRP values and leukocyte values for the combined dependent variables such as blood cultures, fever, and B-symptoms.

**Table 3:** Number of comorbidities and 5-year mortality.

Comorbidities	*n* (%)	5-year mortality
Diabetes mellitus	54 (34.8%)	38.2%
Congestive heart failure	59 (38.1%)	40.8%
Peripheral vascular disease	13 (8.4%)	33.3%
Dementia	5 (3.2%)	40.0%
COPD	10 (6.5%)	30.0%
Liver disease	15 (9.7%)	41.7%
Malignancy	14 (9.0%)	50.0%
Peptic ulcer disease	8 (5.2%)	50%
Renal disease	51 (32.9%)	48.6%
Rheumatic disease	4 (2.6%)	25.0%
Clinical sepsis syndrome	34 (22%)	46.9%

### 3.2 Risk factors for the in-hospital mortality of VO patients

**Figure 3:**
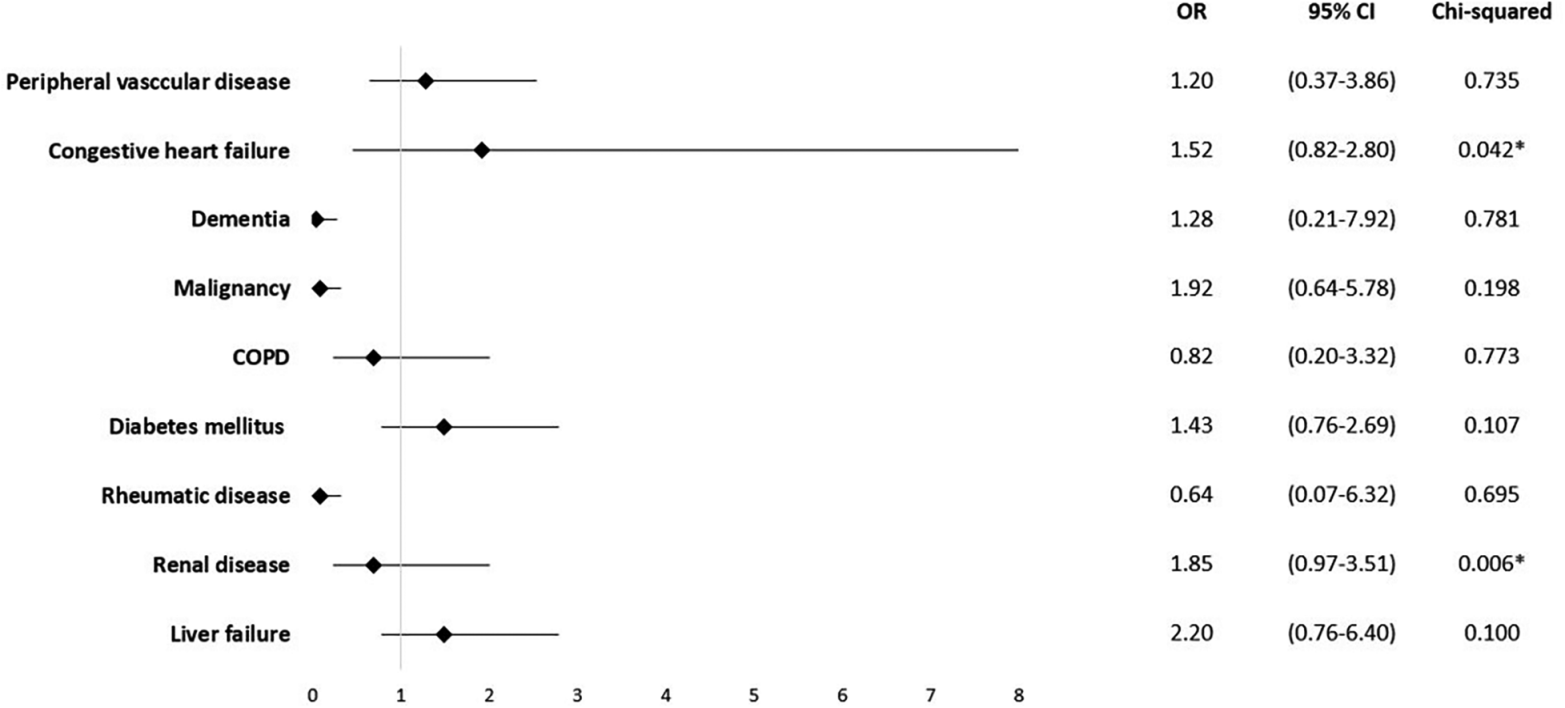
Mortality odds ratio for comorbidities.

Certain underlying health conditions were found to be associated with in-hospital mortality. Renal disease was identified as a risk factor (OR = 1.85; 95% CI 0.97–3.51; *p* = 0.006), as well as congestive heart failure (OR = 1.52; 95% CI 0.82–2.80; *p* = 0.042) (Figure 3).

There was no statistically significant correlation found between mortality and CCI (*ρ*=−0.05; *p* = 0.387) or ACCI (*ρ*=−0.07; *p* = 0.805).

**Figure 4:**
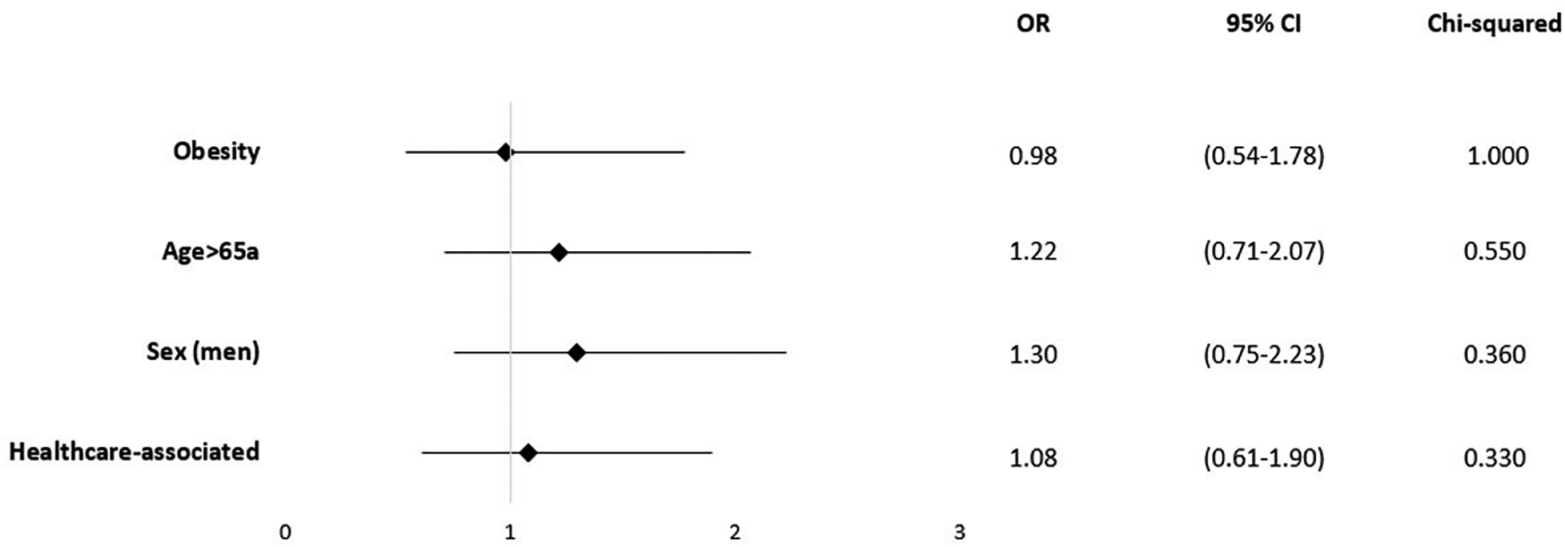
Mortality odds ratio for epidemiological and environmental factors.

In line, obesity (OR = 0.98; 95% CI 0.54–1.78; *p* = 1.00) and age over 65 years (OR = 1.25; 95% CI 0.732.12; *p* = 0–.418) were not significantly associated with in-hospital mortality (Figure 4). Based on the patient records, 78 cases were classified as HAVO (50.3%), while 77 cases (49.7%) were categorized as CAVO. Neither HAVO nor CAVO cases demonstrated an increased in-hospital mortality rate (Figure 4).

Clinical sepsis was found to have a significant association with mortality (OR = 2.44; 95% CI 1.15–5.18; *p* = 0.003). In the cohort, in which blood cultures had been taken the following symptoms were significantly more often documented compared to the cohort without blood cultures: Fever >38°C (*p* = 0.034), clinical sepsis (*p* < 0.001), paravertebral abscess (*p* = 0.003), urinary tract infection (*p* = 0.025), intra-abdominal infection (*p* = 0.008), vascular access infection (*p* = 0.021) and endocarditis (*p* = 0.006). There was no correlation between dental infection, PJI, skin and subcutaneous tissue infection, epidural infection, and psoas abscess.

Pathogens were identified, with *S. aureus* (*n* = 45; 29%) and “other unidentified pathogens” (*n* = 19; 12.3%) and *CoNS* (*n* = 15; 9.7.%) being the most common. In both cohorts, *S. aureus* was the most frequently identified pathogen (46.8% CAVO vs. 51.2% HAVO). This was followed by *CoNS* (20% CAVO vs. 80% HAVO; *p* = 0.016). All cases of vascular access infection were attributed to HAVO (*n* = 5; *p* = 0.024).

**Figure 5:**
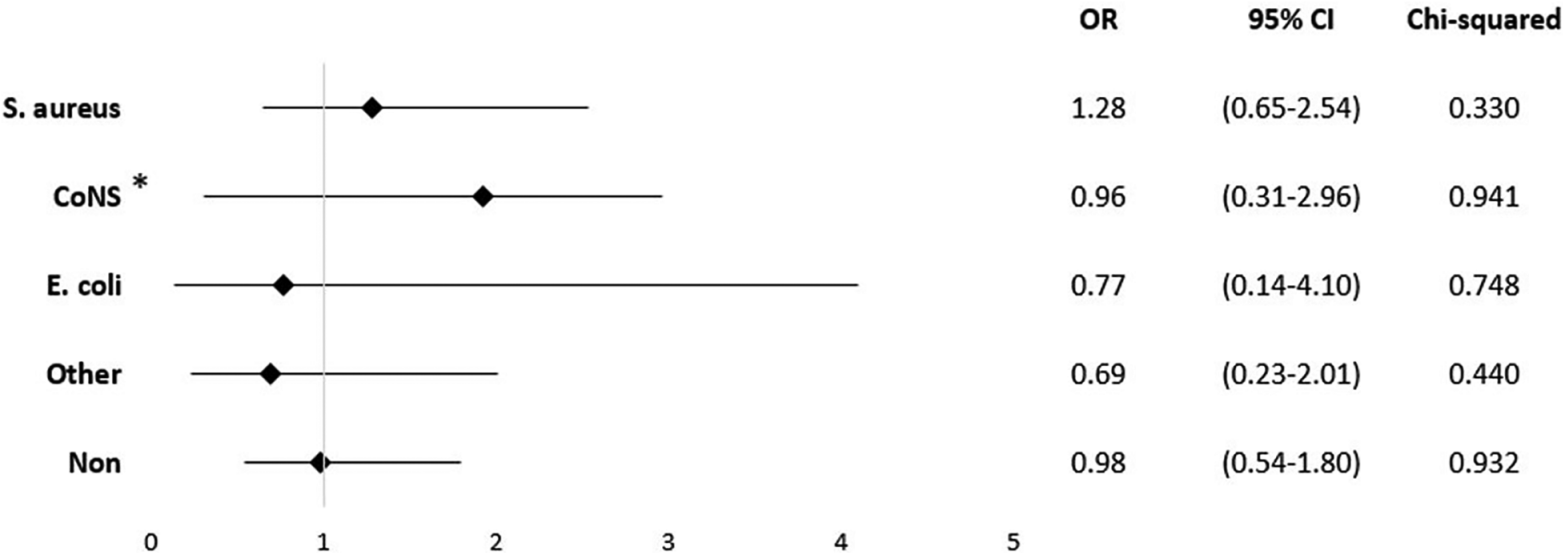
Mortality odds ratio for pathogens. *All other staphylococci without *S. aureus*, for example *S. epidermidis*, *S. haemolyticus*, and *S. saprophyticus*.

When examining the impact of these pathogens, documented infection with *S. aureus* showed the highest risk of mortality (OR = 1.28; 95% CI 0.65–2.54; p = 0.333), although it did not reach statistical significance (Figure 5). There was no correlation between the detection of pathogens and the presence of positive blood cultures. In our cohort, only in cases with *S. aureus* infections psoas-abscess were documented (*p* = 0.023).

## 4 Discussion

The present case series demonstrates that VO is linked with a high rate of in-hospital mortality and a substantial 5-year mortality rate. Regarding outcomes, the 5-year survival rate was 71.7%, with an overall mortality of 28.3% at five years. Patients with congestive heart failure (38.1%), renal disease (32.9%), paraplegia symptoms (10.3%), and sepsis (22%) had notably higher mortality rates. In summary, the study detailed significant associations between surgical intervention and specific conditions, highlighted risk factors for mortality, and emphasized the impact of certain pathogens on patient outcomes in VO cases.

### 4.1 Midterm survival of patients with VO

The overall mortality rates in our patient cohort were 17.2% at one year, 19.9% at two years, and 28.3% at five years. The 5-year-survival rate was 71.7%. Comorbidities that contribute to frailty were linked to an increased risk for mortality in patients with VO. Kidney disease (OR = 1.85) was identified as a high-risk factor as well as congestive heart failure (OR = 1.52).

The observed mortality rate of this study is consistent with the findings reported in existing literature. Mortality from VO has been described with discrepancy ranging from 11% to 20% at one year (15, 28–30). Yagdiran et al. found that the mortality rates at 1 year and 2 years were 20% and 23%, respectively (31). Meanwhile, Vettivel et al, in their single-center study involving 76 patients with pyogenic VO, reported a 5.2% mortality rate at 30 days and a 22.3% mortality rate at 1 year (32). In a multi-center study concentrating on spine surgery, it was evident that infectious diseases substantially increased the risk of in-hospital mortality (33).

### 4.2 Risk factors for the in-hospital mortality of VO patients

Kehrer et al. (28) identified several significant risk factors in their study. They associated severe neurological deficits on admission and the presence of an epidural abscess with a higher risk of short-term mortality. Consistent with our findings, they observed that patients with symptoms of paraplegia had a higher mortality rate. The most commonly types of abscesses, identified in this study were psoas abscesses (27.1%), followed by paravertebral abscesses (12.3%), and epidural abscesses (10.3%). Interestingly, in our study the location of abscesses, regardless of their level, did not significantly affect mortality.

Some studies focused on risk factors of in-hospital mortality after VO. Ziarko et al. (13) analysed risk factor of 9,753 VO cases in Germany in 2020. Apart from age, several comorbidities were associated with an elevated risk of mortality. Notably, heart failure (OR = 2.80) and chronic kidney disease (OR = 1.83) were identified as significant risk factors for mortality. Among the complications studied, clinical sepsis syndrome (OR = 5.94), was associated with increased in-hospital mortality. These results are similar to the current study. Another study by Lenz et al. showed a significant correlation between preoperative glomerular filtration rate (GFR) values with in-hospital mortality in patients with spondylodiscitis (34). A matched case-control study by Kushioka et al. using a multicenter database examined 26.604 patients that underwent spine surgery at 27 Japanese institutions between 2012 and 2018. They identified preoperative renal comorbidity as a significant risk factor for increased mortality (33). Joerger et al. focused on the trend of early mortality among spondylodiscitis patients despite receiving treatment. Among the 430 patients included in the study, a distressing 7.4% (*n* = 32) passed away during their hospital stay. Advanced age, Diabetes mellitus, Previous steroid medication, CCI, and GFR (Glomerular Filtration Rate) level at admission were significant risk factors for in-hospital death (35). Vettivel et al., identified frailty (OR = 13.62) and chronic renal failure (OR = 13.40) as factors associated with a higher risk of 30-day mortality (32). In summary, chronic renal disease is recognized as a well-documented risk factor in the literature. The utilization of contrast media in CT scans and its potential nephrotoxic effects may be one factor contributing to the elevated mortality rates.

Notably, in our analysis, age over 65 years (OR = 1.25) did not reach statistical significance for elevating the risk for mortality. Liver disease showed a clear association (OR = 2.20) but without significancy. In the literature, age and liver disease have already been described as risk factors for mortality (13, 21, 32, 36–38). The mean age in our study is comparable with these studies. However, the mean age at the time of death in our study is lower. Specifically, in our study the mean age in the mortality group was 67.7 ± 12.1 years, whereas in the study by Heuer et al., was 73.8 ± 9.9 years.

Another significant risk factor for increased mortality was the presence of sepsis, which was linked to a 2.44-fold rise in in-hospital mortality. Sepsis is widely recognized for its high mortality rates (39). A systematic review and meta-analysis highlighted a notably elevated mean sepsis mortality rate, reaching 24.4% at 30 days and 32.2% at 90 days (40). Furthermore, sepsis can lead to acute renal failure (AKI), a condition affecting roughly 40%–50% of AKI patients (41). Kidney diseases also contribute to overall mortality, as evidenced in this study.

Sepsis-related acute kidney injury is a frequently encountered complication in critically ill patients, and it is associated with significant health complications and death. Conversely, acute kidney injury has been recognized as a risk factor for sepsis and its associated negative outcomes. In the cohort of patients, from which blood cultures were taken symptoms of complicated clinical courses were significantly more often seen, compared to the cohort without this diagnostic measurement: Fever >38°C, sepsis, paravertebral abscess, urinary tract infection, intraabdominal infection, vascular access infection, and endocarditis. This finding indicated, that blood cultures in our clinical practice have been predominantly obtained in patients with severe symptoms, such as fever, sepsis, or abscesses. However, this underscores the need for a more comprehensive diagnostic approach that includes obtaining blood cultures from afebrile patients as well, to improve pathogen identification and treatment outcomes in cases of VO. Bacterial detection in cases of spondylodiscitis has been reported to be successful in approximately 50%–83% of cases (42). Blood culture significantly enhanced diagnostic accuracy, highlighting the importance of a systematic diagnostic approach. Therefore, it is important to ensure that blood cultures are taken.

Another interesting aspect of VO is the pathogens that have been identified. The most frequently identified pathogens were *S. aureus* (29%), followed by the *S epidermidis* (5%) as already known in the literature (20, 42, 43). The notable predominance of other *staphylococci* strongly indicated a significant presence of *CoNS*. This is consistent with recent findings on the increasing importance of HAVO, particularly those associated with devices that cause bloodstream infections and contribute to the development of spondylodiscitis (44, 45). Furthermore, in our study, spondylodiscitis with *CoNS* detection demonstrated a significantly higher incidence in the HAVO group compared to the CAVO group. A retrospective study involving 586 cases of spondylodiscitis identified that *S. aureus* was predominantly present in individuals younger than 60 years old. On the other hand, gram-negative bacteria were more prevalent among those aged over 60 years. Additionally, there was an association observed between the presence of gram-negative pathogens and patients diagnosed with solid tumors or liver cirrhosis (46). Michels et al. highlighted the significant antimicrobial resistance profile seen in *CoNS*, particularly in healthcare environments (47). Due to *CoNS*’ lower virulence in comparison to *S. aureus* in VO (48), these infections might frequently go unnoticed and receive delayed diagnoses. Intravascular catheter-related infections are considered responsible for up to 34% of HAVO VO cases (45). Another study described *S. aureus* infections, the presence of gram-negative pathogens with higher mortality (13, 36, 43). In our cohort, infection with *S. aureus* as the only pathogen demonstrated a significantly higher risk of psoas abscess, a complication that could contribute to a severe course of the disease. Interestingly, Priest et al. (49) reported that 60% of all cases with *S. aureus* detection either progressed to developing an epidural, paraspinous, or psoas abscess (44).

### 4.3 Operative treatment

Surgical debridement stands as a widely acknowledged treatment for infectious diseases. Its primary goal is to reduce the spread of infection, speed up the control of the infection, and at the same time, provide tissue samples that could help adjust additional antibiotic treatment (50).

In a meta-analysis that involved 21 studies and a total of 10.954 patients, researchers compared early surgical intervention to conservative management. The analysis evaluated that opting for early surgery showed a correlation with decreased mortality rates (8% vs. 13% in conservative treatment) and lower rates of relapse/failure (15% vs. 21%). Furthermore, early surgical intervention led to a notably shorter hospital stay, mean 7.8 days. These consistent findings provide strong evidence in favor of prioritizing early surgical management for cases involving pyogenic spondylodiscitis (51).

Similar to our findings, a systematic review included 64 studies revealed that 54.7% of cases underwent surgical treatment. Moreover, a substantial majority of cases involving neurological complications (83.9%, totaling 120 cases) underwent surgery (52).

Literature indicates that there is evidence favoring surgical intervention, particularly in cases of cervical spondylodiscitis compared to thoracolumbar conditions. Cervical infections have been associated with notably higher morbidity and mortality rates. Often cervical spondylodiscitis is linked to neurological symptoms (53, 54). For instance, in one cohort of 15 cases with cervical spondylodiscitis, 67% of patients displayed neurological deficits at the time of diagnosis (55), with similar findings in a study of 19 patients at a different medical center (56). Contrary to the established literature, this study did not find an association between cervical spondylodiscitis and the presence of neurological symptoms. All in all, patients presenting with paraplegia, paralysis, sepsis and back pain underwent general surgical treatment more frequently. Blecher et al. initiated non-surgical therapy for 35 patients diagnosed with vertebral osteomyelitis. They suggested that fever and a history of intravenous drug abuse and fever as well as the extent of osseous and posterior element involvement could serve as valuable indicator advocating for early surgical intervention in the treatment of spinal infections (57).

Regarding limitations, it is important to note that VO may not only depend on the identified risk factors, but could be influenced by several other factors not measured in this study. The assertion that VO is only influenced by the known risk factors is difficult, as there may be additional variables at the same time that still need to be investigated. Furthermore, similar risk factors for other infections such as PJI appeared in this study. It is significant that these identified risk groups may need customised therapies and adaptations. However, it's important to note that this study has certain limitations. The retrospective design of this study, coupled with its small sample size and single-center scope, limits the generalizability of the findings, as it may not adequately capture the diverse clinical presentations and management strategies in a broader patient population. A notable constraint is that the occurrence of positive blood culture results, may be attributed to other sources of infection (such as infected central venous catheters, pneumonia, etc.). In this retrospective study, blood cultures were apparently obtained mainly from patients with severe symptoms, potentially skewing results to suggest a higher correlation between positive cultures and severe disease courses. Additionally, it should be noted that the standardized protocol for blood culture collection is currently lacking, warranting further attention in clinical practice. The lack of pathogen identification in patients not subjected to blood cultures hinders targeted antibiotic therapy, underscoring the need for more systematic blood culture usage across varying symptom severities in VO cases.

## 5 Conclusion

In conclusion, the evaluation of individual patient risk factors, including the presence of comorbidities, is essential for the effective management and treatment of VO. Our findings underscore the necessity of vigilant monitoring, especially for VO patients with chronic organ disease, swift identification, and management of sepsis, as well as the prioritization of accurate pathogen detection and antibiotic sensitivity testing, to enhance survival prospects in this vulnerable patient cohort.

## Data Availability

The raw data supporting the conclusions of this article will be made available by the authors, without undue reservation.
